# PD-L1 regulates tumorigenesis and autophagy of ovarian cancer by activating mTORC signaling

**DOI:** 10.1042/BSR20191041

**Published:** 2019-12-20

**Authors:** Hongmin Gao, Juan Zhang, Xiaohong Ren

**Affiliations:** 1 Department of Obstetrics and Gynecology, Xi’an Fourth Hospital, Xi’an, Shaanxi 710004, China; 2 Department of Obstetrics, Affiliated Hospital of Yan’an University, Yan’an, Shaanxi 716000, China; 3 Department of Gynecology, Shaanxi Nuclear Industry 215 Hospital, Xianyang, Shaanxi 712000, China

**Keywords:** autophagy, mTORC, ovarian cancer, PD-L1

## Abstract

PD-L1 is a well-known immune co-stimulatory molecule that regulates tumour cell escape from immunity by suppressing the immune response. However, the clinical significance of PD-L1 in the progression of ovarian cancer is unclear. Our study demonstrated that PD-L1 is up-regulated in ovarian tumour tissue compared with its expression level in adjacent normal tissue. Furthermore, we confirmed that PD-L1 increases the proliferation of cancer cells by activating the AKT-mTORC signalling pathway, which is also enhanced by the expression of S6K, the substrate of mTORC. In addition, PD-L1 promotes the autophagy of ovarian cancer cells by up-regulating the expression of BECN1, a crucial molecule involved in the regulation of autophagy. In conclusion, PD-L1 may provide a target for the development of a novel strategy for the treatment of ovarian cancer.

## Introduction

Ovarian cancer (OC) is one of the most common malignant tumours in women, and its mortality rate ranks first among gynaecological tumours [1,2]. Many risk factors contribute to the development of OC, such as obesity, oestrogen level, and genetic alterations. The survival of OC patients is still unsatisfactory despite greatly improved therapeutic methods [3]. Once OC reaches an advanced stage, the treatment effectiveness is eventually limited, and new treatments are urgently needed. Unfortunately, the means of inhibiting tumour proliferation and increasing the survival rate are not completely clear.

The PI3K–AKT signalling pathway is crucial for regulating many cellular functions, which it does by modulating the phosphorylation, transcription and translation of the downstream targets necessary for catabolic and anabolic processes [4–8]. Preclinical studies demonstrated that AKT is activated frequently in OC, resulting in over-activation of AKT signalling cascades, including migration or invasion, proliferation, apoptosis, survival and metabolic functions [9–11]. mTORC1 regulates protein production and cell metabolism, directly phosphorylating ribosomal S6 kinase (S6K) and promoting protein synthesis; S6K controls the translation of several mRNAs that encode many protein components [12–15]. Phosphorylation of S6K1 (P-S6K1) is a key biomarker of functional mTOR pathway activation and is abnormally expressed in prostate cancer, melanoma, breast cancer, lung cancer and colorectal cancer [16–19].

Recent studies have shown that immunosuppression provides an opportunity for cancer to progress [20–24], and some immunosuppressive factors, such as PD-L1, play important roles in this process [25]. PD-L1 has reportedly been involved in regulating the progression of multiple cancers, including melanoma, breast and hepatocellular cancer. In melanomas, the expression of PD-L1 within tumours was considered a prognostic marker [26,27], and treatment with PD-L1 antibodies in hepatocellular carcinoma reduced the escape of liver tumour cells [28]. Moreover, PD-L1 expression is a prognosis factor for poor outcomes in soft-tissue sarcomas [29], and the frequent expression of PD-L1 in inflammatory breast cancer indicates that it has a weak response to chemotherapy [30]. In the present study, the expression levels and potential roles of PD-L1 on human OC were evaluated. The results illustrated that the expression levels of PD-L1 were remarkably up-regulated in human OC tissue specimens compared with that of adjacent normal tissues. Furthermore, overexpression of PD-L1 significantly strengthened OC cell proliferation. These results might provide reliable evidence for the development of new therapeutics for human OC treatment.

## Materials and methods

### Tissue sample collection

Ovarian cancer samples were obtained from Shaanxi Nuclear Industry 215 Hospital. Written informed consent was obtained from the patients, and the study was approved by the Institutional Human Experiment and Ethics Committee of Shaanxi Nuclear Industry 215 Hospital. The Institutional Review Board (IRB) approval number is 2017SNIH-034.

### Cell culture and treatment

The ES2 cell line was purchased from ATCC (CRL-11731) and maintained in DMEM supplemented with 10% FBS and 1% antibiotic–antimycotic solution (100 U/ml penicillin and 100 μg/ml streptomycin). Transfection was performed with Lipofectamine TM 2000 (Invitrogen, Carlsbad, CA, U.S.A.) according to the manufacturer’s instructions.

### Chemicals

IFN-γ (Cat. # IFG-H4211) was purchased from ACROBiosystems (CA, U.S.A.). MK-2206 (Cat. #HY-10358) and WYE-687 (Cat.# HY-15271) were purchased from MCEy (Shanghai, China).

### Cell viability assay

Cells were seeded at a density of 3000 cells per well in 96-well culture plates and treated with a series of IFN-γ concentration levels for a specific duration or administered at different time points. After treatment, the cells were incubated with fresh media containing CCK-8 at 37°C for 3 h. Then, the absorbance was measured at 570 nm and the results were recorded by an enzyme-linked immunosorbent assay reader.

### EdU proliferation assay

To assess the level of cell proliferation, ES2 cells were seeded in 96-well plates. The cells were incubated in complete media under standard conditions. After transfection, the cell assays were performed on specific days, as mentioned above. Forty-eight hours after transfection, cell proliferation was measured by incorporating 5-ethynyl-2′-deoxyuridine (EdU) with an EdU cell proliferation assay kit (Solarbio, Beijing, China). Briefly, the cells were incubated with 40 µM EdU for 5 h and then fixed, permeated and stained with EdU according to the manufacturer’s protocol. The nuclei were stained with DAPI (Sigma) at a concentration of 1 µg/ml for 30 min. The proportion of the cells incorporated with EdU was measured with fluorescence microscopy.

### Western blotting

The protein was extracted with lysis buffer [20 mM Tris (pH 7.5), 150 mM NaCl, 1% Triton X-100, sodium pyrophosphate, β-glycerophosphate, EDTA, Na_3_VO_4_, and 1 mM leupeptin cocktail]. Thirty micrograms of protein were loaded onto 10% polyacrylamide gels for sodium dodecyl sulfate-polyacrylamide gel electrophoresis (SDS-PAGE). The gel was transferred onto a nitrocellulose membrane, which was blocked with 5% non-fat dry milk for 1 h at room temperature and then incubated with primary antibodies overnight at 4°C.

After washing with TBST, the membrane was incubated with a secondary antibody for 1 h at room temperature. The signalling was detected with an enhanced chemiluminescence HRP substrate by Western blotting (Pierce, Rockford, IL, U.S.A.). The following primary and second antibodies were purchased from Proteintech (Wuhan, CHINA): PD-L1 (#28076-1-AP, 1 to 1000 dilution), S6K (#14485-1-AP, 1 to 2000 dilution), BECN1 (#11306-1-AP, 1 to 2000 dilution), p-AKT (# 66444-1-Ig, 1 to 1000 dilution), AKT (#10176-2-AP, 1 to 1000 dilution), LC3 (#18725-1-AP, 1 to 2000 dilution), GAPDH (# 10494-1-AP, 1 to 5000 dilution), HRP-linked anti-rabbit secondary antibody (#10256-2-AP, 1 to 5000 dilution) and HRP-linked anti-mouse secondary antibody (#10358, 1 to 5000 dilution). P-S6K (# 9204S, 1 to 1000 dilution) were purchased from Cell Signaling Technology, Inc (CST, U.S.A.).

### Real-time PCR

Total RNA was extracted from human ovarian tumour tissues using TRIzol reagent (TaKaRa, Dalian, China). Reverse transcription was performed by using HiScript Reverse Transcriptase following the manufacturer’s instructions (Vazyme, Nanjing, China). Three micrograms of total RNA was used for the reverse transcription reaction. Quantitative PCR was performed using SYBR Green Real-Time PCR Master Mix (Vazyme, Nanjing, China). All values of sample expression were normalized to that of the glyceraldehyde-3-phosphate dehydrogenase (GAPDH) (the internal reference control). The following PCR protocol was used: 5 min at 95°C, followed by 32 cycles at 95°C for 15 s, 56°C for 15 s and 72°C for 1 min. The ∆∆*C*q method was used to process the data and calculate the relative gene expression. The following human primer sequences were used: PD-L1 forward: 5′-CAAATGTTGTTTGGGTCATGC-3′ and reverse: 5′-GTAAAACGACGGCCAGTCATTCCTTCCTCTTGTCACGC-3′ and GAPDH forward: 5′-GGCATGGACTGTGGTCATGAG-3′ and reverse: 5′-TGCACCACCAACTGTTAGC-3′.

### IHC staining

The core tissue biopsy sample exhibiting carcinoma with a 2.5-mm diameter was punched from individual donor paraffin-embedded tissue blocks and arranged accurately into a new recipient block. A sample of 4 μ was cut and used for IHC analysis. PD-L1 expression was detected via immunohistochemical staining using streptavidin peroxidase. Briefly, the slices were de-waxed with xylene and rehydrated through an ethanol gradient wash. The endogenous peroxidase activity was blocked with 3% hydrogen peroxide in methanol for 15 min. After antigen retrieval in 10 mmol/l citrate phosphate buffer for 5 min, the sections were blocked in 10% normal goat serum for 1 h. The sections were then incubated with PD-L1 antibody at 4°C overnight. Vectastain® Elite ABC kits (Peroxidase) (Vector Laboratories, Burlingame, CA, U.S.A.) were used for the detection of PD-L1 signalling.

### Statistical analysis

All results were confirmed through replication of at least three independent experiments, and all quantitative data are presented as the mean ± SD. Student’s *t* test or one-way ANOVA was used to analyse quantitative variables. The Kaplan–Meier method was used to evaluate survival curves, and the log-rank test was used to test the differences between the survival curves. A result was considered statistically significant when a bilateral *P*-value was less than 0.05.

## Results

### PD-L1 is overexpressed in human ovarian cancer and predicts a poor outcome

**Figure 1:**
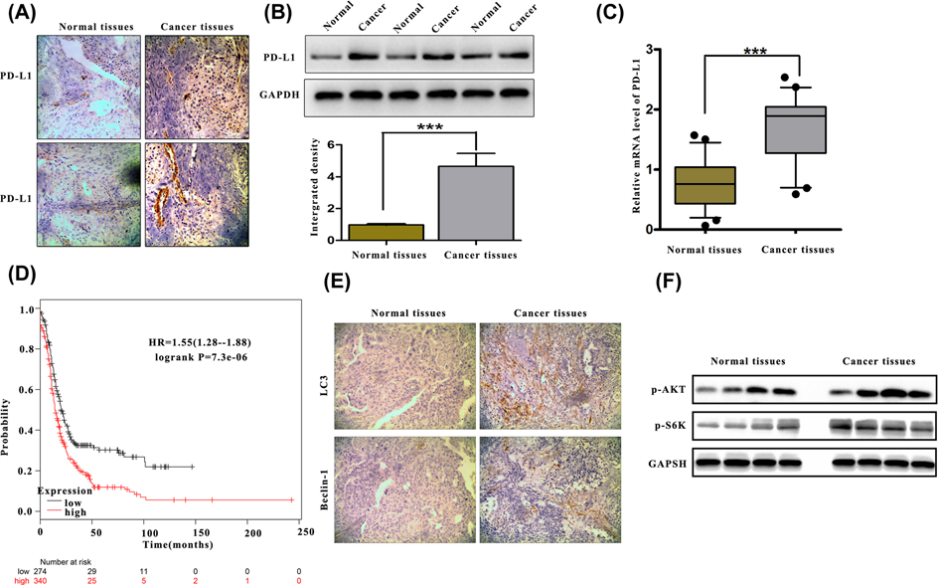
Increased expression of PD-L1 in human ovarian cancer (**A**) Immunohistochemical (IHC) staining against the PD-L1 protein in ovarian cancer tissue and adjacent normal tissue. (**B**) The expression of PD-L1 was measured by Western blotting in ovarian tumours and normal adjacent tissues. (**C**) RT-PCR was used to detect PD-L1 mRNA expression in five representative pairs of the indicated tissues. (**D**) Kaplan–Meier plot analysis showed that the survival of OC patients depended on the expression level of PD-L1. *P*-values were determined using the log-rank test, and *P* ≤ 0.05 was considered significant. (**E**) IHC staining against the LC3 and Beclin-1 protein in ovarian cancer tissue and adjacent normal tissue. (**F**) The expression of p-AKT and p-S6K were measured by Western blotting in ovarian tumours and normal adjacent tissues. ***P<0.001.

To investigate the influence of PD-L1 on human ovarian cancer progression, IHC was performed to examine the protein expression level of PD-L1 in five cases of paraffin-embedded ovarian tissues. PD-L1 expression was significantly increased in primary tumour tissues compared with adjacent normal tissues (ANT) (Figure 1A), supporting the potential relationship between ovarian carcinoma proliferation and PD-L1 expression. Consistently, PD-L1 expression was markedly overexpressed in the ovarian cancer tissues compared with paired normal tissues at the protein and mRNA levels (Figure 1B,C). To determine the clinical relevance of PD-L1 in ovarian cancer, the Kaplan–Meier survival analysis and log-rank test were used, and the results showed that PD-L1 overexpression was correlated with lower overall survival (Figure 1D). These results suggest that PD-L1 expression is clinically relevant to the survival of OC patients.

### The proliferation of OC cells was increased after stimulation with IFN-γ

**Figure 2:**
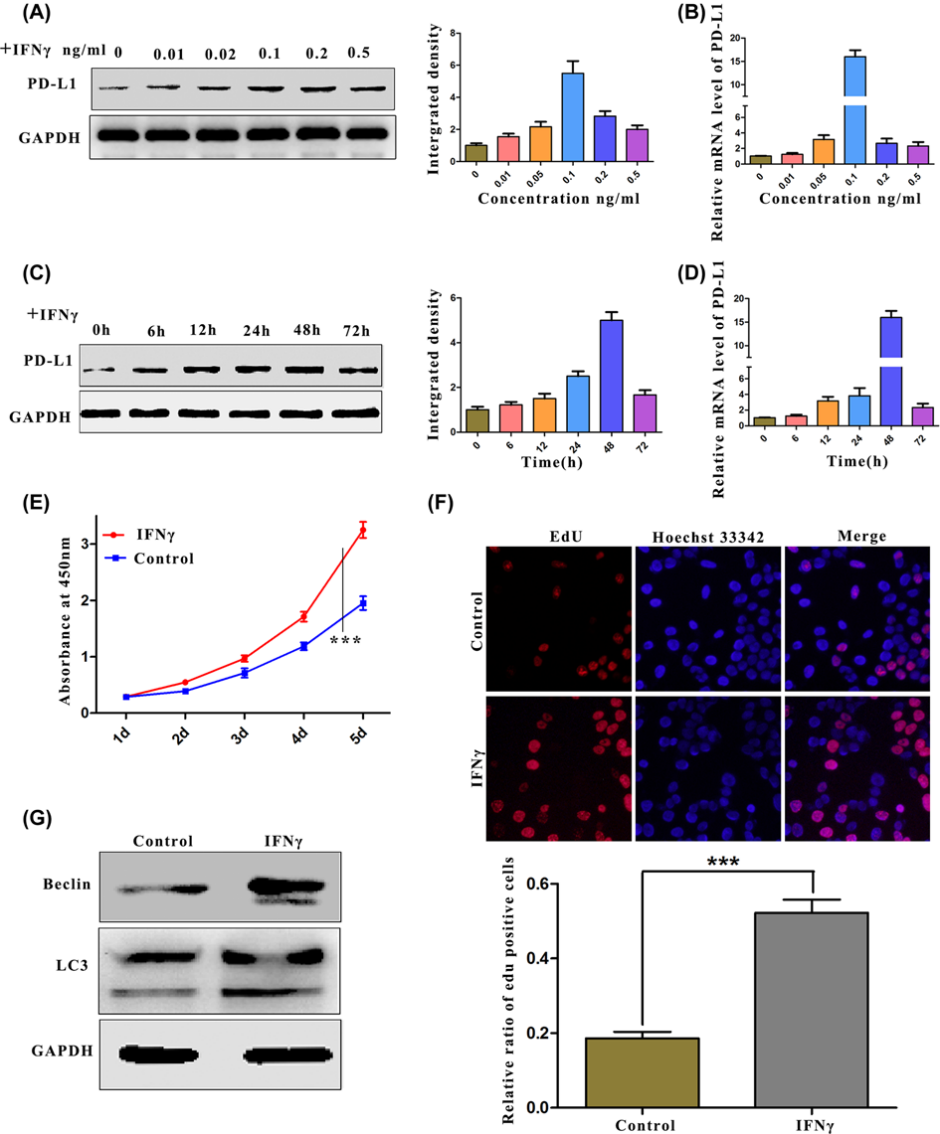
Stimulation of IFN-γ increased ovarian cancer cell proliferation (**A**–**D**) The effect of IFN-γ stimulation on PD-L1. (A) PD-L1 protein expression after stimulation with different concentrations of IFN-γ was measured by Western blotting. (B) The mRNA expression of PD-L1 after stimulation with IFN-γ at different concentrations was measured by RT-PCR. (C) The protein expression of PD-L1 after stimulation with IFN-γ at different time points was measured by Western blotting. (D) The mRNA expression of PD-L1 after stimulation with IFN-γ at different time points was measured by RT-PCR. (**E** and **F**) Effects of IFN-γ on the proliferation of ovarian cancer cells. (E) ES2 cells were treated with IFN-γ (50 ng/ml). The viability of the cells was determined by CCK-8. (F) ES2 cells were treated with IFN-γ (50 ng/ml) for 48 h. The viability of the cells was determined by EdU staining. (**G**) ES2 cells were treated with IFN-γ (50 ng/ml) for 24 h. LC3 expression was measured by Western blotting. *P*-values were determined using the log-rank test, and *P* ≤ 0.05 was considered significant.

To further explore the biological role of PD-L1 on the progression of human ovarian carcinoma, we used a cell model in which the up-regulated expression of PD-L1 was stimulated by IFN-γ [31–33]. In these experiments, we first detected the expression of PD-L1 by Western blotting and real-time quantitative PCR after the cells were stimulated with IFN-γ. The experimental results showed that, compared with that of the general ES2 cells, PD-L1 protein expression was up-regulated dramatically with a prolonged stimulation time or changed IFN-γ concentration (Figure 2A–D). On this basis, we screened the biological changes in the tumours after IFN-γ stimulation. We evaluated the effects of PD-L1 on ES2 cell proliferation using the CCK-8 assay. The proliferation of cells stimulated by IFN-γ was significantly increased (Figure 2E). To further determine the biological effect of PD-L1 on the proliferation of ovarian cancer cells, the EdU assay was used to detect the effect of PD-L1 on cell proliferation. Consistent with the results of the CCK-8 assay, the percentage of EdU-positive cells was significantly higher in cells stimulated by IFN-γ compared with the percentage in the control cells (Figure 2F). According to relevant reports in the literature, there is an integral relationship between autophagy and cell proliferation, and PD-L1 reportedly participates in the regulation of autophagy. Therefore, in the present study, the effects of autophagy were detected by Western blotting. After IFN-γ stimulation of the cells, the experimental results proved that autophagy was clearly activated in human ovarian cancer cells, as represented by the increase in accumulated LC3II (Figure 2G). In summary, the up-regulation of PD-L1 not only promotes the proliferation of ovarian cancer cells, but also regulates cell autophagy.

### The stimulation of IFN-γ influences the proliferation of cancer cells and depends on PD-L1

**Figure 3:**
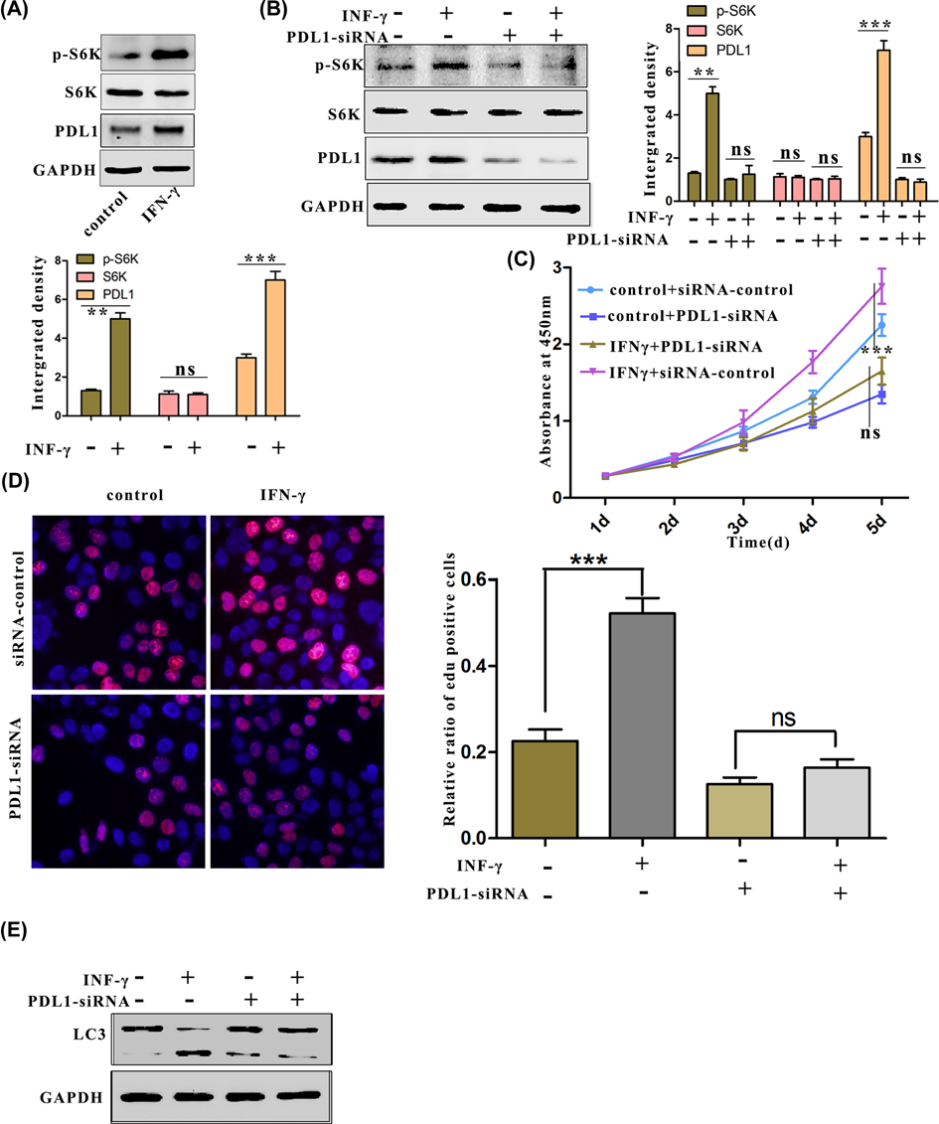
Increased proliferation of OC by the stimulation of IFN-γ depends on PD-L1 (**A**) After stimulation for 24 h, the phosphorylation of S6K was measured by Western blotting. (**B**) S6K phosphorylation after the indicated treatment was measured by Western blotting. (**C** and **D**) The proliferation of ES2 cells after the indicated treatment was measured by CCK-8 and EdU staining. (**E**) ES2 cells transfected with siPD-L1 after stimulation for 12 h. LC3 protein expression was determined by Western blotting.

Previous studies have reported that the mTORC1 pathway plays an important role in regulating cell autophagy and proliferation [13,34,35]; we examined whether stimulated PD-L1 promotes cell proliferation through the mTORC1 signalling pathway by assessing the expression of S6 kinase 1 (S6K1), which is a downstream phosphorylation substrate molecule of mTORC1. The results from the Western blot analysis showed that the IFN-γ stimulation up-regulated the phosphorylation of S6K1 (Figure 3A), whereas the phosphorylation of S6K1 was not increased in PD-L1-knockdown cells (Figure 3B). To determine whether IFN-γ-induced PD-L1 is crucial in IFN-γ-enhanced ovarian cancer cell proliferation, a PD-L1-specific RNAi was used to down-regulate the endogenous overexpression of PD-L1. In addition, the CCK-8 assay demonstrated that the cell proliferation was enhanced after the IFN-γ stimulation but that it was not enhanced in the PD-L1-deficient cells (Figure 3C). Consistent with these results, in ES2 cells depleted of endogenous PD-L1, fewer EdU-positive cells were observed compared with the number found among the control cells (Figure 3C). In addition, knocking down PD-L1 also inhibited the autophagy that had been induced by IFN-γ stimulation (Figure 3D). The above results demonstrate that the increased phosphorylation of S6K, cell proliferation, and autophagy that was induced by IFN-γ stimulation depended on PD-L1.

### The AKT signalling pathway contributes to the increased proliferation of IFN-γ stimulated ovarian cancer cells

**Figure 4:**
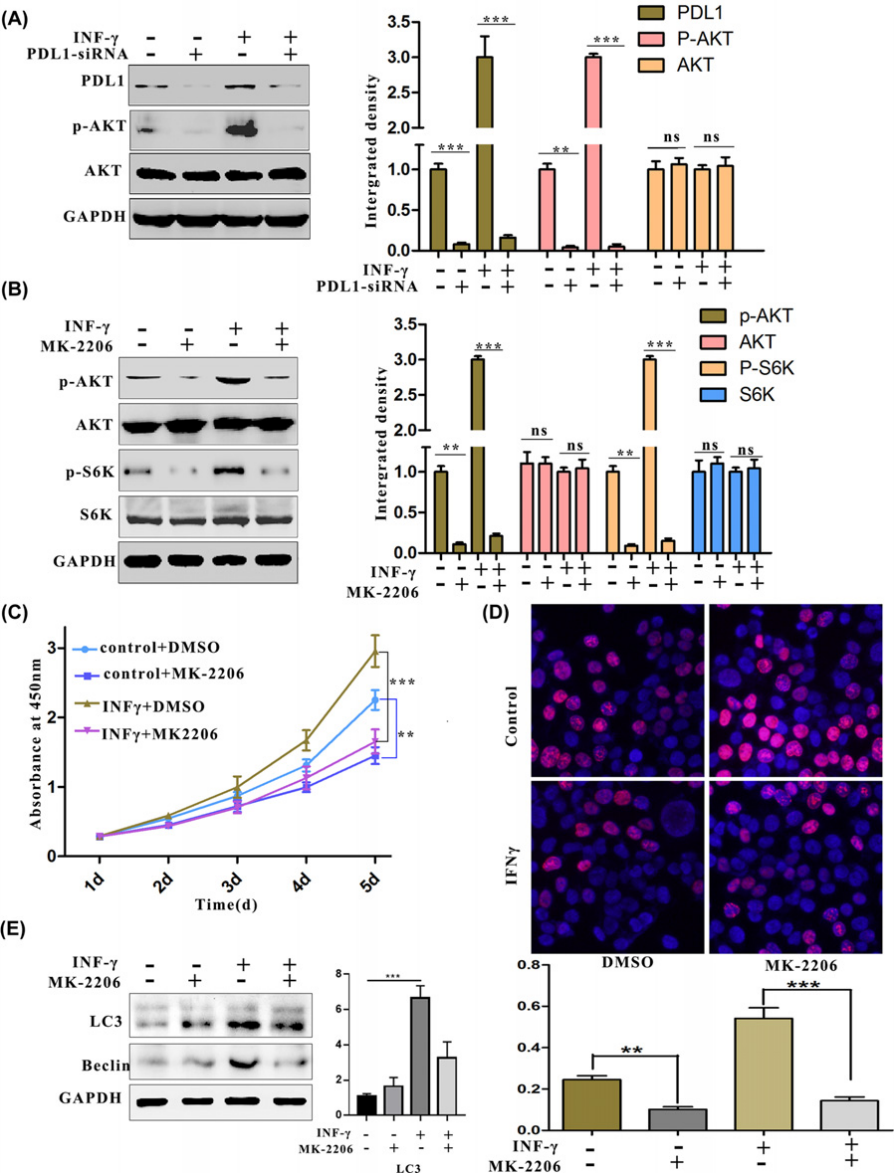
Increased proliferation of OC by the stimulation of IFN-γ through the activation of the AKT signalling pathway (**A** and **B**) Quantification of phosphorylated AKT. (A) The cells were treated with IFN-γ (50 ng/ml) for 24 h in the presence of PD-L1 siRNA. AKT phosphorylation was measured by Western blotting. (B) The cells were treated with IFN-γ (50 ng/ml) for 24 h in the presence of MK-2206. The phosphorylation of AKT and S6K was measured by Western blotting. (**C** and **D**) Results from the quantification of OC cell proliferation. (C) The viability of ES2 cells after treatment with IFN-γ (50 ng/ml) for 24 h in the presence of MK-2206 was measured by CCK-8. (D) The proliferation of ES2 cells after treatment with IFN-γ (50 ng/ml) for 24 h in the presence of MK-2206 was determined by EdU staining. (**E**) LC3II protein expression was measured after the indicated treatment. **P<0.01.

Overactivation of the PI3K–AKT cascade is one of the most common events in human cancer progression [36–40]. To further explore the mechanism by which PD-L1 affects ovarian cell proliferation, we hypothesized that the AKT pathway participated in the effects of PD-L1 on tumour cell proliferation. Western blotting showed that IFN-γ stimulation up-regulated the phosphorylation of AKT, whereas knocking down PD-L1 attenuated the hyperactivation of the AKT signalling pathway that had been induced by IFN-γ (Figure 4A). To further verify that stimulation by IFN-γ regulated AKT, an inhibitor of the AKT pathway was used to detect whether it could reverse the effects of IFN-γ stimulation. The results from the Western blot analysis verified that the stimulation of MK-2206 blocked the phosphorylation of AKT that had been stimulated by IFN-γ (Figure 4B). Moreover, the CCK-8 and EdU assays showed that the up-regulated proliferation of ovarian carcinoma cells was suppressed after stimulation with MK-2206 (Figure 4C,D). Additionally, experiments demonstrated that the AKT inhibitor also inhibited autophagy by down-regulating the expression of beclin1, a key autophagy regulator (Figure 4E). In summary, the proliferation and autophagy of ovarian cells by the stimulation of IFN-γ depend on AKT signalling pathways.

### The activated mTORC pathway collaborates with PD-L1 to regulate the proliferation of cancer cells

**Figure 5:**
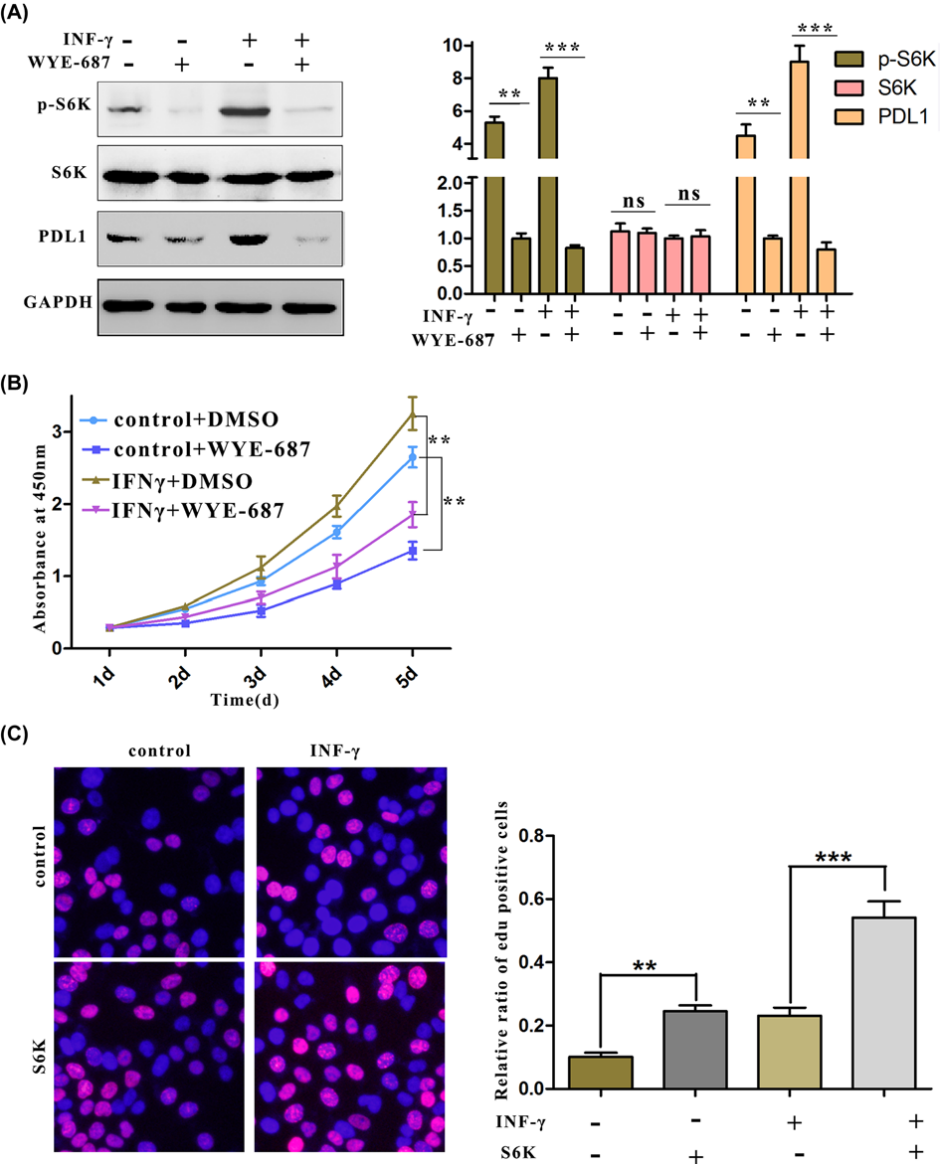
The mTORC pathway plays an important role in regulating the viability of OC cells (**A**) After the cells were treated with IFN-γ (50 ng/ml) for 24 h in the presence of WYE-687(20 nM), PD-L1 protein levels were measured by Western blotting. (**B** and **C**) Results from the quantification of OC cell proliferation. (B) The viability of ES2 cells after treatment with IFN-γ (50 ng/ml) for 24 h in the presence of WYE-687 (20 nM) was measured by CCK-8. (C) The proliferation of ES2 cells after treatment with IFN-γ (50 ng/ml) for 24 h during S6K overexpression was measured by EdU staining.

Previous experiments demonstrated that PD-L1 not only affects autophagy in ovarian carcinoma by activating the AKT–mTORC pathway, but also increases cell proliferation by inducing the phosphorylation of S6K. Therefore, we hypothesized that S6K, the kinase that regulates proliferation and protein synthesis, has an impact on PD-L1 expression, and the results from Western blotting proved that the inhibition of mTORC significantly reduced the protein levels of PD-L1 (Figure 5A). Furthermore, cancer cell proliferation was also inhibited by the use of the mTORC inhibitor; in contrast, overexpression of S6K, the downstream substrate of mTORC (Figure 5B), further enhanced the proliferation of cancer cells that had been stimulated by IFN-γ (Figure 5C). In summary, these results indicated that PD-L1 and factors in the mTORC signalling form a positive pathway that promotes OC cell proliferation.

## Discussion

OC is one of the most common malignant tumours in women and ranks first in mortality among gynaecological tumours [41,42]. Therefore, it is necessary to determine the molecular mechanisms responsible for the progression of OC. In addition, the findings will be valuable for developing new therapeutic targets and strategies. Previous studies have shown that PD-L1 is involved in the progression of several types of human malignancy. Increased PD-L1 levels were reverse-correlated with poor outcomes in breast and colorectal cancer [43]. PD-L1 was reported to facilitate metastasis in pancreatic cancer [44]. However, an inverse correlation existed between the survival and expression of PD-L1 in early-stage non-muscle-invasive bladder cancer [45]. In our study, we observed increased expression of PD-L1 in primary OC tissues compared with normal tissues. The data showed that PD-L1 could increase the proliferation of ovarian cancer cells.

Collectively with the other report, the ribosomal protein S6K (S6 kinase) represents an extensively studied effector of the target of rapamycin complex 1 (TORC1), which plays important roles in cellular and organismal physiology [46,47]. The mTORC1–S6K1 axis controls basic cellular processes that are necessary for the growth of organisms, including transcription, biosynthesis, cell growth and metabolism [48–51]. Some of the factors in this pathway have reportedly acted as cancer-promoting gene in serval malignant tumours. For example, S6 kinase promotes OC cell metastasis, and recent research showed that activation of mTOR/S6K enhanced the proliferation of HER2-breast cancer [52]; S6K also participated in regulating the proliferation of prostate cancer. In addition, Komatsu et al. found that S6K could change the sensitivity of KRAS-mutant cancer cells to ERK inhibitors [53], and our results demonstrate that S6K cooperates with PD-L1 to induce the up-regulation of S6K. These findings further indicate PD-L1 as a possible target that enhances tumour proliferation in ovarian cancer.

BECN1 is a well-established regulator of autophagy, and Beclin-1 could be affected by a number of molecules in the cell, which can lead to either activation or inhibition of autophagy [54–57]. TRIM59 regulated autophagy by modulating both the transcription and the ubiquitination of beclin1, while AMPK regulated autophagy by phosphorylating BECN1. In addition, ABHD5 could also interact with BECN1 to regulate the autophagy and tumorigenesis of colon cancer cells [58]. Here, we indicated that overexpression of PD-L1 enhances the protein level of BECN1. These results demonstrate that PD-L1 is involved in the regulation of autophagy independent of the mTORC pathway.

In summary, the present study identifies the regulatory link between PD-L1 and mTORC/S6K and describes a potential mechanism explaining PD-L1, which contributes to ovarian cancer growth and cell proliferation. Our study demonstrates that the expression of PD-L1 is significantly up-regulated in clinical OC tissues and is positively associated with the phosphorylation of S6K1. Furthermore, PD-L1 and S6K constitute a pathway that affects the proliferation of cancer cells. Therefore, PD-L1 has important value as an indicator of progression in OC patients and may become a therapeutic target in the future.

## Abbreviations

EdU: 5-ethynyl-2′-deoxyuridine
GAPDH: glyceraldehyde-3-phosphate dehydrogenase
OC: ovarian cancer
P-S6K1: phosphorylation of S6K1
