# Expression of Concern: PD-L1 regulates tumorigenesis and autophagy of ovarian cancer by activating mTORC signaling

**DOI:** 10.1042/BSR-20191041_EOC

**Published:** 2021-04-07

**Keywords:** autophagy, mTORC, ovarian cancer, PD-L1

The Editorial Office has been made aware of potential issues surrounding the scientific validity of this paper, hence has issued an expression of concern to notify readers whilst the Editorial Office investigates.

